# Beyond Gir: Barda wildlife sanctuary as a potential stronghold for Asiatic lions

**DOI:** 10.1371/journal.pone.0354096

**Published:** 2026-09-09

**Authors:** Mohan Ram, Aradhana Sahu, Nityanand Srivastava, Rohit Chaudhary, Lahar Jhala, Yashpal Zala

**Affiliations:** 1 Gujarat Forest Department, Government of Gujarat, Gandhinagar, Gujarat, India; 2 Department of Wildlife Sciences, Banda University of Agriculture and Technology, Banda, Uttar Pradesh, India; Amity University, INDIA

## Abstract

The expansion of Asiatic lions (*Panthera leo persica*) (lions) beyond their core area and the recolonisation of Barda Wildlife Sanctuary (Gujarat) after 144 years—through natural dispersal of a male and augmentation of five females by the Gujarat Forest Department—make Barda’s conservation and management crucial for the species’ long-term survival. We assessed the space use, habitat preferences, social organisation, and diet of Asiatic lions in Barda using radio-collaring (n = 6; 1 male, 5 females) and scat analysis. The average lion home range (95% KDE) was 70.55 ± 7.48 km², while the average core area (50% KDE) was 10.92 ± 1.92 km². Spatial overlap among individuals was higher at the home range level (0.60 ± 0.08) than in core areas (0.34 ± 0.06), indicating spatial partitioning and emerging social structuring. Lions demonstrated hierarchical habitat selection, preferring mixed forests at the home range scale and grasslands within core areas. Dietary analysis showed a strong reliance of lions on wild prey, particularly wild pigs (*Sus scrofa*; 55% occurrence), supplemented by domestic cattle (17%). Ongoing prey augmentation efforts, including the reintroduction of spotted deer (*Axis axis*) and sambar (*Rusa unicolor*), aim to strengthen prey availability in the Barda. Our findings provide ecological insights suggesting that Barda offers potentially suitable habitat and resources for sustaining a long-term lion population, although these inferences are based on a limited sample size and short-term dietary assessment. Proactive management interventions focusing on habitat restoration, prey-base enhancement, and human–lion conflict mitigation will be essential to consolidate Barda as a secure and enduring conservation stronghold.

## Introduction

Due to their extensive movements, predatory behaviour, and charismatic appeal, large carnivores act as umbrella, keystone, and flagship species, making them crucial to biodiversity conservation and ecosystem functioning [1–3]. Despite such ecological importance, large carnivore populations have suffered alarming declines worldwide, primarily due to human-induced activities such as habitat loss, human-wildlife conflict, and poaching [4,5]. These risks have necessitated science-based conservation strategies and policy interventions to stabilise population declines and secure the long-term survival of large carnivores. However, some carnivore species, for example, the Asiatic lion (*Panthera leo persica*), tiger (*Panthera tigris tigris*) and wolves (C*anis lupus*) have demonstrated remarkable recovery when effective conservation measures and favourable policies are implemented [6–8].

Recolonising carnivore populations face several challenges as they spread beyond their core habitats, including habitat fragmentation, increased anthropogenic presence, and competition for resources [9–12]. This underscores the importance of identifying and managing suitable habitats outside core habitats that may act as ecological sinks, providing vital resources and connectivity for dispersing carnivore populations [13,14]. Among large carnivores, one of the most remarkable conservation success stories is that of the Asiatic lions. Historically ranging from the Middle East to northern India, Asiatic lions are now distributed across about 35,000 km^2^ in the Saurashtra peninsula of Gujarat, India [7]. Focused and concerted conservation efforts have led to significant recovery, with the latest population estimate reporting 891 individuals [7]. The Gir National Park & Wildlife Sanctuary (hereafter, Gir) in Gujarat, India, has served as the sole stronghold and source population for the Asiatic lion [7]. This conservation achievement has led to natural density-dependent dispersal, resulting in nine distinct satellite populations in the surrounding landscape of Gir, which function as population buffers for the growing population [7]. Such expansion can be understood within the framework of metapopulation dynamics, where spatially structured populations are linked through dispersal, and source–sink dynamics, in which core areas like Gir act as sources while newly colonising habitats may initially function as sinks before attaining demographic stability. These satellite populations require detailed ecological information not only about the species itself but also regarding the habitats they occupy to support conservation and management efforts better. Such knowledge provides a strong foundation for developing targeted management strategies to enhance habitat quality, support prey-base populations, and reduce human-wildlife conflict, thereby ensuring the long-term survival of lions beyond their core habitat.

The Barda Wildlife Sanctuary (hereafter Barda) in Gujarat has emerged as an important habitat for supporting the growing Asiatic lion population [7]. Historically, lions inhabited Barda until 1879, and the natural dispersal of a male lion to the sanctuary on 19 January 2023 reaffirmed its importance as a functional corridor and conservation site. To support re-establishment, five female lions of various age classes were subsequently released into Barda. Barda has the potential to serve as a future source population for lions dispersing further into the southwestern landscape of Saurashtra, Gujarat. However, for Barda to function as a viable habitat for a source population, effective conservation measures must be undertaken using a sound scientific approach. Crucial ecological factors such as space-use patterns, habitat selection, prey availability and diet, social organisation, and interactions with surrounding land-use patterns are vital for informed management. Currently, the only scientific information available from Barda relates to prey species availability [15], while data on other ecological aspects of lions are lacking.

The present study was conducted with the aim of conserving and managing lions in Barda, Gujarat, India, with the following objectives: (a) to assess the home range, habitat preferences, and interactions among radio-collared lions; (b) to evaluate the diet of lions; (c) to suggest conservation and management aspects in Barda.

In the case of home ranges, we hypothesized that the average home range of a lion would be larger than that of Gir due to the low prey base in Barda [15]. Human disturbances such as livestock grazing, religious pilgrimages, and settlement activities (*nesses* – temporary human settlements of pastoral communities) are primarily concentrated in the flatter terrain. Therefore, we hypothesize that, at the home range level, lions will prefer mixed forest in rugged terrain over habitats in flatter areas and valleys to avoid human disturbance. Additionally, previous studies have shown that lions preferentially hunt large prey; however, the wild ungulate population in Barda is skewed towards medium-sized prey, particularly wild pigs [15]. Therefore, we hypothesize that lions will prefer prey that are numerically abundant.

## Materials and methods

### Study area

**Fig 1:**
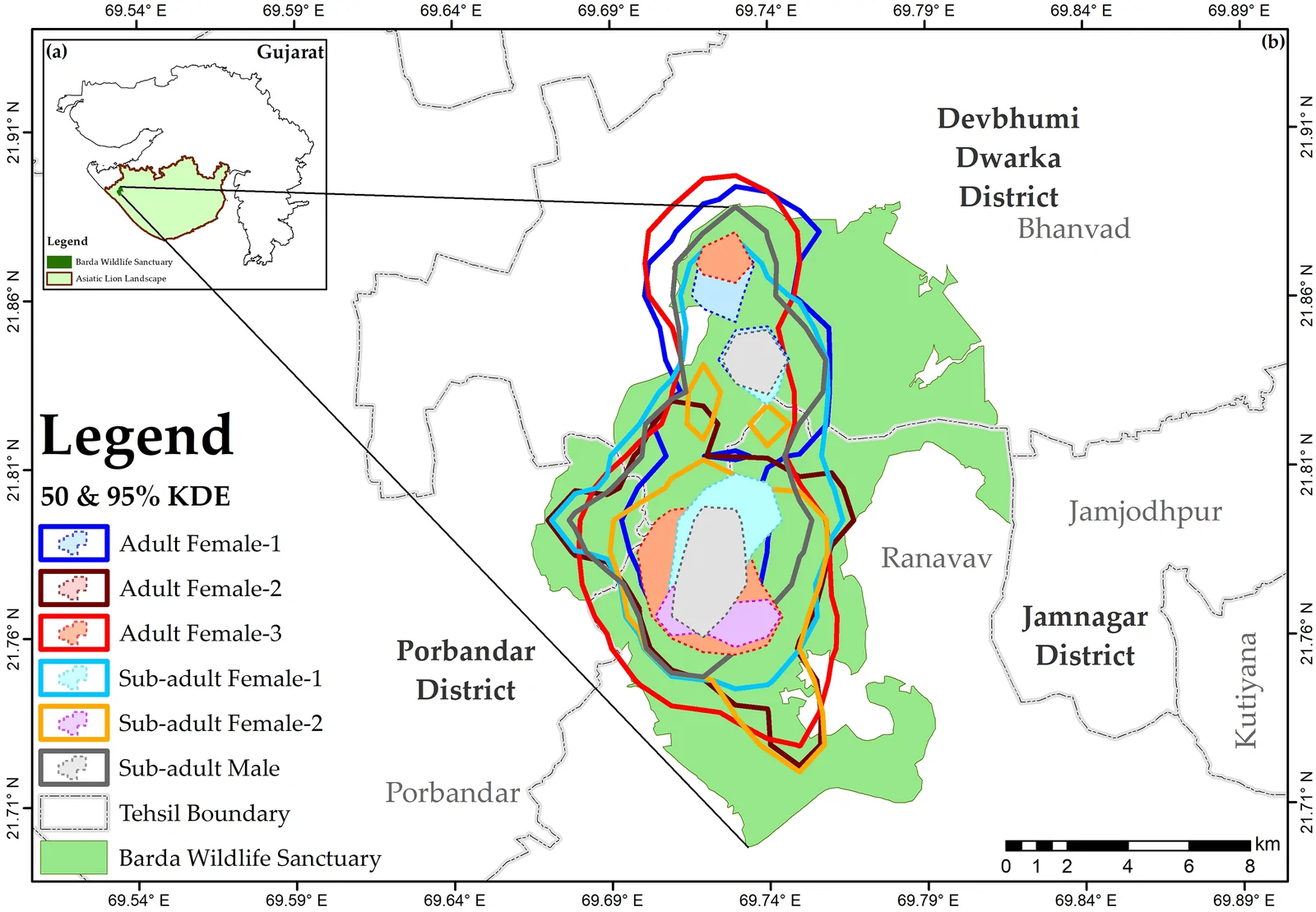
Map of the study area showing the home ranges (95% KDE) and core areas (50% KDE) of individual lions in Barda Wildlife Sanctuary. The age class and current age of each lion correspond to the details provided in Table 1. This figure was generated using ArcGIS 10.8.1 in the Gir Hi-Tech Monitoring Unit, specifically for the purpose of this paper’s study area map, and is CC BY 4.0.

Barda, located between 21°40’N and 21°55’N and 69°40’E and 69°50’E, lies in the Saurashtra region of Gujarat, western India (Fig 1). Covering 192.31 km², the sanctuary features a rugged, hilly landscape with elevations ranging from 79.2 m to 617.8 m above sea level [15] (Fig S1 in S1 File). It falls within a semi-arid biogeographic zone and hosts various forest types, such as 5A-Southern tropical forest, 5A/C3-Southern dry mixed deciduous forest, and 6B-Northern tropical thorn forest [16]. Dense forest covers 16.3% of the sanctuary, open forest accounts for 29.9%, and the remaining area is classified as degraded forest [17] (Figs S1 and S2 in S1 File). The region experiences three distinct seasons: summer (March to June), with peak heat in May and June when temperatures reach 42°C; monsoon (July to October), with an average annual rainfall of approximately 650 mm; and winter (November to February), where December and January are the coldest and driest months, with nighttime temperatures dropping to 8–10°C [18]. Two prominent rivers, Kileshwari and Minsar, flow through the sanctuary. Barda WLS supports rich biodiversity, harbouring 368 floral species, including 59 trees, 83 shrubs, 200 herbs, and 26 climbers [19]. Its faunal diversity includes 22 mammals, 269 birds, 26 reptiles, 4 amphibians, and 55 butterfly species [20]. The vegetation is characterised by diverse associations, such as *Acacia nilotica – Acacia senegal, Dichrostachys cinerea, and Wrightia tinctoria – Butea monosperma* [20].

**Table 1:** Details of Asiatic lions monitored in Barda Wildlife Sanctuary.

Sr. No.	Code of Individual	Age Group and Sex	Age (at the time of radio-collaring)	Current Age (as on December 2025)	Data Monitoring Period		Number of Monitoring Days	Number of Locations
					From	To		
1	SAM-1	Sub-adult Male	3-5 years	6-8 years	19/01/2023	30/07/2025	896	51143
2	SAF-1	Sub-adult Female	4-5 years	7-8 years	31/03/2023	30/07/2025	770	28018
3	AF-1	Adult Female	9-10 years	11-12 years	09/08/2023	30/07/2025	680	31971
4	AF-2	Adult Female	5-9 years	7-11 years	27/02/2024	30/07/2025	363	16326
5	AF-3	Adult Female	5-9 years	7-11 years	27/02/2024	30/07/2025	434	19523
6	SAF-2	Sub-adult Female	3-5 years	5-7 years	27/02/2024	30/07/2025	501	22192
**Total**								**169173**

### Methodology

#### Capture and radio-collaring of lion individuals.

We deployed Vertex Plus GPS radio collars (Vectronic Aerospace GmbH, Berlin, Germany) on six individual lions, ensuring the collar weight remained below 3% of each lion’s body weight, regardless of age or sex (Table 1). We radio-collared all adult lions (n = 6), while the remaining individuals (n = 11) were dependent on adult females. Thus, we achieved 100% coverage of the adult population, enabling assessment of space use and interaction patterns in this re-establishing population. For immobilisation, we used a combination of ketamine hydrochloride (2.2 mg/kg body weight; Ketamine, Biowet Puławy sp. z o.o, Poland) and xylazine hydrochloride (1.1 mg/kg body weight; Xylaxil, Brilliant Bio Pharma Pvt Ltd, Telangana, India), administered using a gas-powered Telinject™ G.U.T 50 dart delivery system (Telinject, Inc., Dudenhofen, Germany). Standard body measurements were recorded for each immobilised individual in accordance with the Gujarat Forest Department’s standard operating procedures (SOP). After collar deployment, we injected Yohimbine hydrochloride (0.1–0.15 mg/kg body weight; Yohimbe, Equimed, Allentown, NJ, USA) intravenously to reverse the sedation, enabling full recovery within 5–10 minutes [21]. All radio-collaring procedures were performed by experienced veterinarians and wildlife healthcare teams.

The study was carried out between 19 January 2023 and 30 July 2025 as part of the Asiatic Lion Radio-Telemetry Project. Data from the radio-collared individuals were gathered and monitored by the Gir Hi-Tech Monitoring Unit, a technology-driven scientific monitoring initiative based in Sasan-Gir, Gujarat. Each collar was equipped with a mortality sensor, a programmable drop-off feature, and a GPS schedule recording the lion’s location every hour.

To assess habitat use, we employed a used-availability design at two spatial scales: the home range scale (2^nd^ order) and within the home range scale (3^rd^ order). The study considered six habitat types: *Acacia* Forest, Grassland, *Euphorbia* Scrub, Mixed Forest, Scrubland, and Water bodies (Fig S1 in S1 File). Water bodies were incorporated to represent the available habitat in their surrounding areas (Fig S3 in S1 File). Second-order habitat use was assessed within the 95% and 50% KDE home range areas for each lion, while habitat availability was defined at the broader Barda landscape scale.

We further evaluate elevation use employing a used-unused sampling design [22]. All GPS locations from collared lions were considered as used points, representing elevations utilised by lions. To characterise the range of accessible elevations to lions, an equal set of random points was generated across the Barda and treated as unused points. Elevation values were then extracted for both used and available locations to create the dataset for analysing elevation use.

#### Diet pattern.

We used scat analysis, an effective, non-invasive method to examine dietary patterns [23]. Lion scats were opportunistically collected along trails and roads, with the date and GPS coordinates recorded for each find. The scat samples were collected between April 2024 and June 2024. Samples that could not be conclusively identified were excluded from the analysis. The samples were cleaned by rinsing them under running water through a fine sieve, then dehydrated in 70% ethanol, and finally dried on filter paper [24]. For prey identification, reference slides were prepared from hair samples collected from the field and from the Sakkarbaug Zoological Park, Junagadh, Gujarat. The prey species were identified by comparing medullary patterns of hair from scats with those of reference slides [25]. In total, 63 lion scats were collected and processed for the analysis.

The Gujarat Forest Department has undertaken the augmentation of two key prey species—spotted deer and sambar—through a combination of captive breeding and translocation. Individuals bred in captivity are released into Barda as part of an ongoing supplementation programme. To further expedite prey recovery, the department has also initiated the translocation of these species from the Gir Protected Area. Both augmentation strategies are currently in progress in Barda, and the systematic records maintained by the department were utilised in the present study.

### Analysis

#### Home range and habitat utilisation.

We used Kernel Density Estimation (KDE) to estimate the home range of radio-collared lions. Since KDE home range analysis could be affected by temporal autocorrelation in the recorded GPS locations and therefore, we selected one random location from each day (07:00–19:00 hours, Indian Standard Time) and night (19:01–06:59 hours, Indian Standard Time). The Least Squares Cross-Validation (LSCV) method was employed to determine the optimal bandwidth for each individual, ensuring a data-driven approach that minimises estimation error [26]. The 95% KDE was used to define the home range, while the 50% KDE assessed the core area [27]. Home ranges were analysed using the adehabitatHR package in R [28].

Subsequently, we evaluated the spatial overlap of home ranges and core areas (95% and 50%) among individual lions using the Volume of Intersection (VI) index, which ranges from 0 (no overlap) to 1 (complete overlap) [29,30]. By analysing the positions and shapes of the utilisation distributions of two radio-collared individuals and calculating the volume of intersection of their three-dimensional home range surfaces, the VI quantifies spatial overlap [31]. The VI index derives a single measure of overlap using the complete utilisation distributions of both individuals.

We used the proximity rate (PR) to examine interactions [32] among the radio-collared lions. The PR measures the proportion of simultaneous fixes at which two individuals approached each other within a specified distance threshold [33]. We employed the PR to assess whether two overlapping lions always avoided using the same space at the same time. We considered one hour as the time threshold for defining simultaneous fixes between two individuals. Additionally, we assumed a 500 m distance threshold to consider two individuals as being in proximity. The selected time interval and distance threshold were informed by practical constraints and adapted from established methodologies in wildlife telemetry studies, including Long (2015) [32] and Dertien et al. (2021) [34]. This index ranges from 0 to 1, with 0 indicating avoidance and values close to 1 indicating attraction.

To measure habitat selection at both the second and third orders, we employed Jacobs’ Index of habitat preference [35]. Jacobs’ Index ranges from −1 to +1, where values close to −1 signify strong avoidance, values near +1 denote strong preference, and values around 0 indicate neutral use of a habitat type [35].

To assess how elevation influenced lion space use, we fitted a quadratic logistic regression model using the used–available dataset [36]. The binary response variable (1 = used, 0 = unused) was modelled against elevation and its quadratic term to capture potential non-linear patterns. A quadratic form was chosen because elevation is expected to impose an energetic cost on large carnivores. As elevation increases, terrain becomes steeper and movement becomes more energetically demanding, so lions are unlikely to exhibit a simple linear increase in use. The quadratic model thus allowed us to detect this ecologically realistic pattern by identifying the elevation range where space use peaks before declining or saturating at higher elevations.

#### Diet patterns, prey availability and augmentation.

Diet composition was analysed using two main metrics: Frequency of Occurrence (FO) and Percent Occurrence (PO) [23]. FO indicates the proportion of scat samples containing a specific prey item, calculated by dividing the number of scats with a specific prey species by the total number of scat samples. PO, meanwhile, represents the percentage of each prey species relative to all prey occurrences across all species. To address the potential overrepresentation of smaller prey species in dietary analyses through FO and PO, biomass consumption was estimated using a generalized model for tropical felids developed by Chakrabarti et al. (2016) [37]. The relative biomass for each prey species was calculated by dividing its biomass contribution by the total biomass consumed. The dietary niche breadth of lions was evaluated using Levin’s index, which ranges from 0 (indicating a specialised diet) to 1 (indicating a generalised diet). Regarding prey availability, no analysis was conducted during the study period; instead, data from a previous study [15] were utilised. The prey augmentation data were assessed based on the pattern of individuals released into the wild in Barda.

## Results

### Home range and habitat utilisation

We obtained a total of 169173 locations of six radio-collared lions from 19 January 2023 to 30 July 2025 (Table 1). The average home range (95% KDE) of lions in Barda was 70.55 ± 7.48 km², while the core area (50% KDE) was 10.92 ± 1.92 km². A two-sample t-test revealed a significant difference between the sizes of home range and core area in Barda lions (t = 2.22, p < 0.05). The largest home range was recorded for AF_3, at 100.20 km², followed by SAF_1 (82.15 km²), SAM_1 (68.90 km²), AF_1 (65.23 km²), AF_2 (58.29 km²), and SAF_2 (48.52 km²) (Fig 1). The largest core area was also for AF_3, at 17.88 km², followed by SAF_1 (15.46 km²), SAM (10.42 km²), AF_2 (8.36 km²), SAF_2 (6.83 km²), and AF_1 (6.60 km²) (Fig 1).

**Fig 2:**
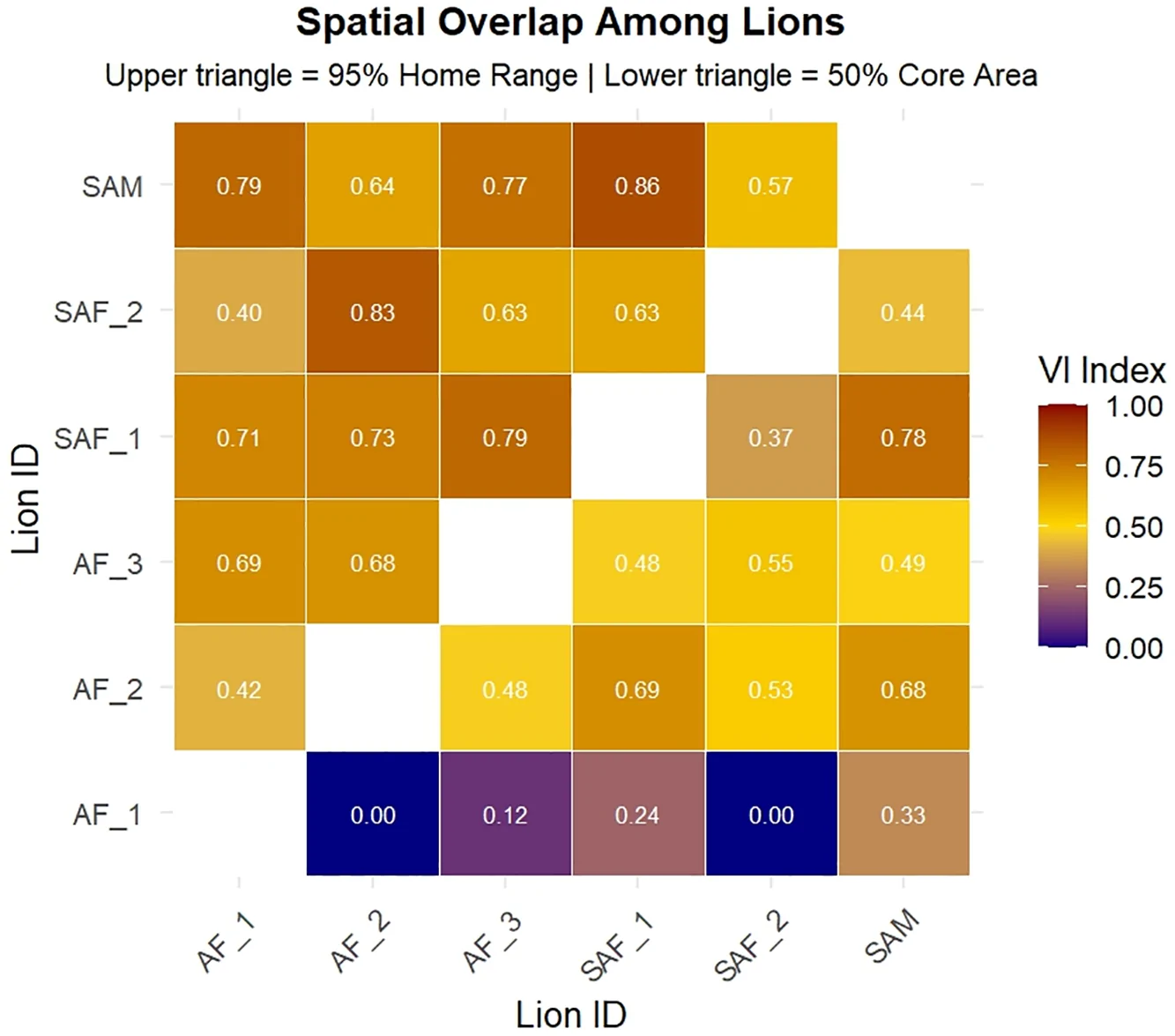
Heat map showing overlap in the home ranges (95% KDE) and core area of radio-collared individuals in Barda Wildlife Sanctuary, Gujarat, India.

The average home range overlap among lion individuals was 0.60 ± 0.08 SE. The highest overlap was observed between SAF_1 and SAM (0.864), while the lowest overlap was recorded between AF_1 and SAF_2 (0.40) (Fig 2).

The average core area overlap among lion individuals was 0.34 ± 0.06 SE. The highest overlap was observed between SAF_1 and SAM (0.78), while the lowest overlap was recorded between AF_1 – AF_2 and AF_1 – SAF_2 (0.000) (Fig 2).

**Fig 3:**
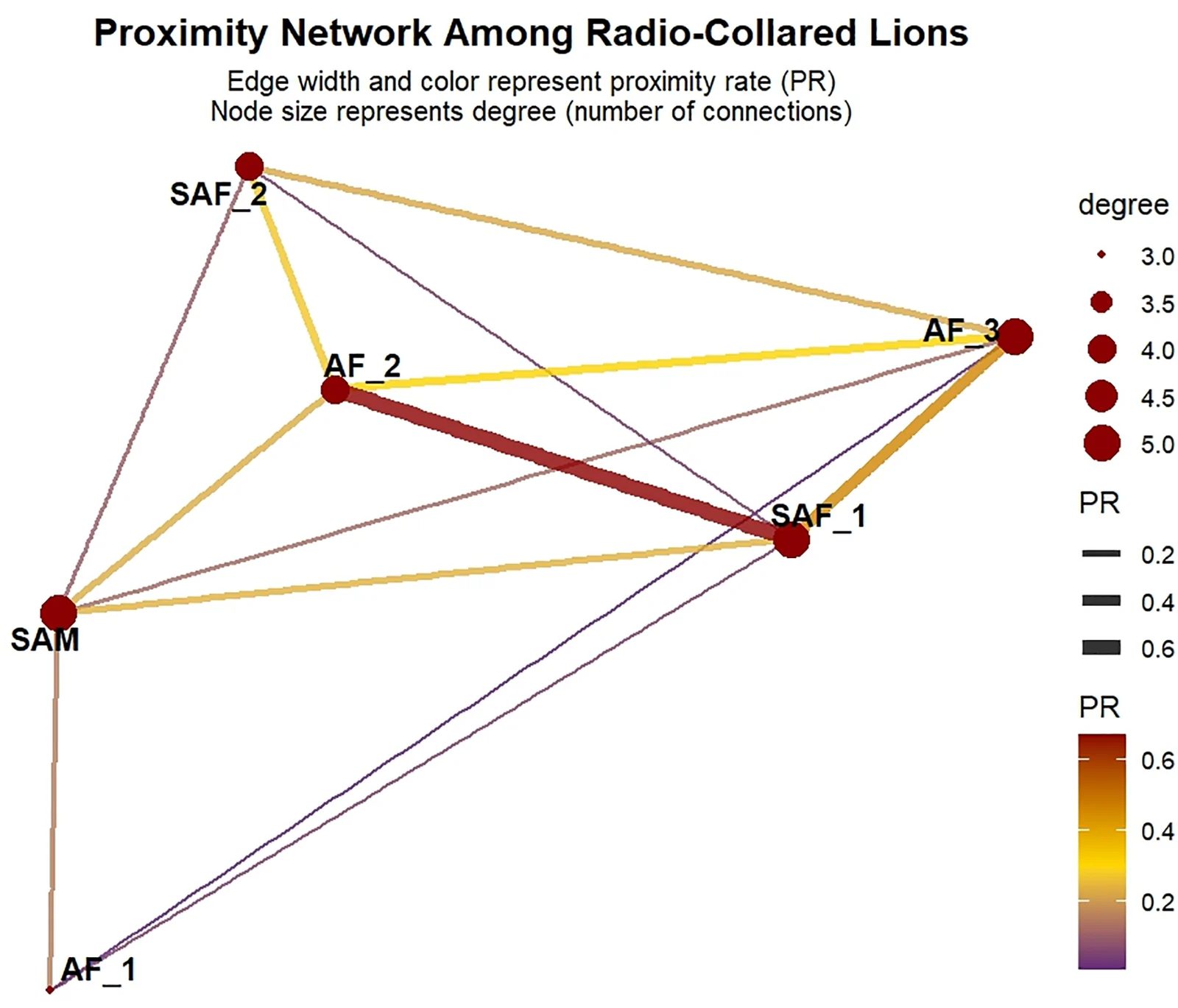
Spatial interaction among the radio-collared lions in Barda Wildlife Sanctuary, Gujarat, India.

Proximity rate (PR) among different lions showed considerable variation, ranging from 0.000 to 0.667, reflecting notable differences in fine-scale spatial association among individuals (Fig 3; Fig S4 in S1 File). The lowest proximity values were observed between AF_1–AF_2 (PR = 0.000) and AF_1–SAF_2 (PR = 0.000), followed by AF_1–AF_3 (PR = 0.007), indicating minimal close-range use between these dyads. In contrast, the strongest proximity was recorded between AF_2–SAF_1 (PR = 0.667), followed by AF_3–SAF_1 (PR = 0.458) and AF_2–AF_3 (PR = 0.307), suggesting greater fine-scale spatial interaction within these pairs (Fig 3).

### Habitat selection

**Fig 4:**
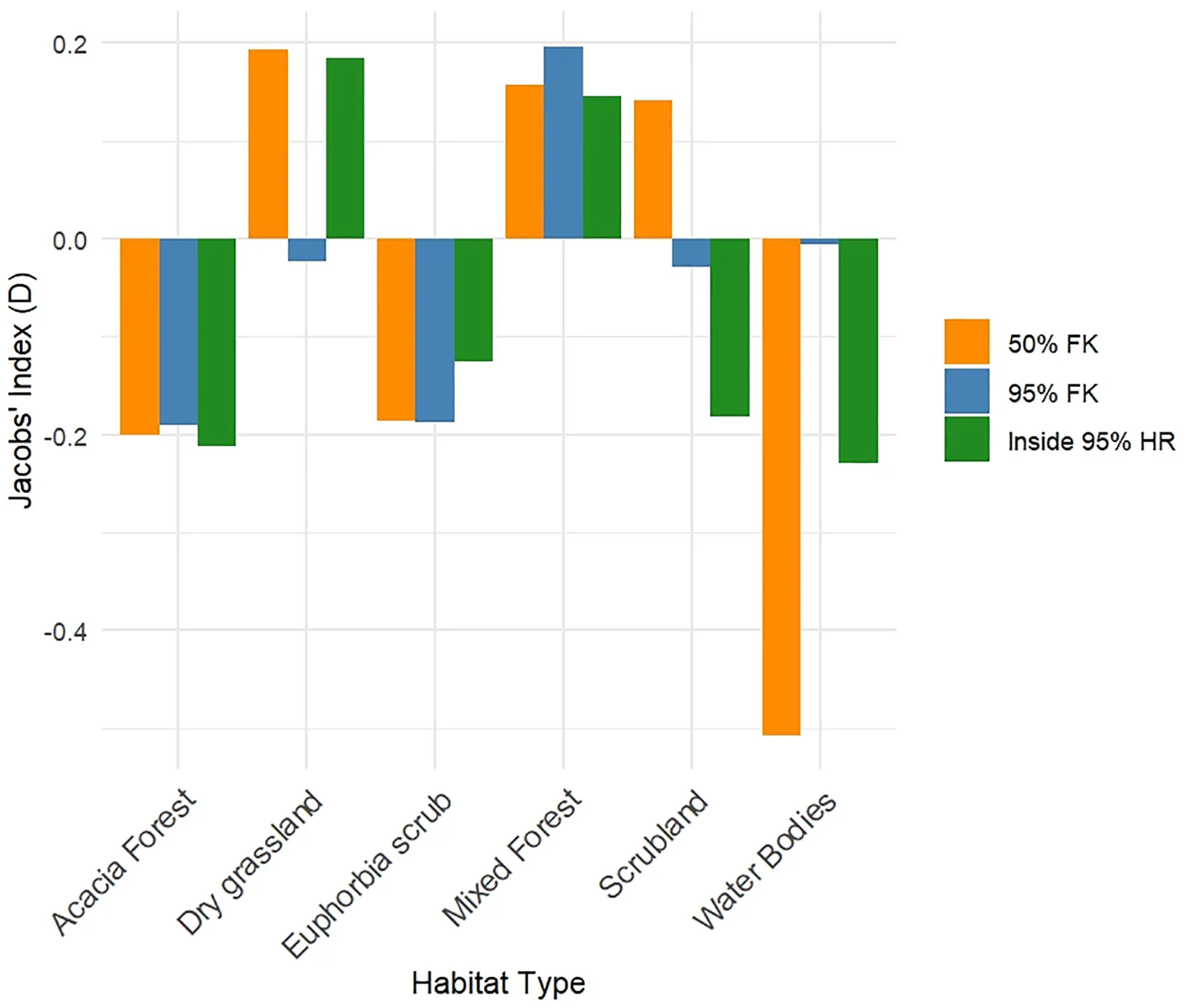
Habitat preference by lions in Barda Wildlife Sanctuary, Gujarat, India.

Jacob’s index showed that at the 2^nd^ order with a 95% KDE level, lions only show preference for the mixed forest (Fig S5 in S1 File) and avoidance for the *Acacia* forest, grassland, *Euphorbia* scrub, and scrubland, while using habitats near water bodies in relation to availability (Fig 4). At 50% KDE, lions show preferences for grassland, mixed forest, and scrubland while avoidance for the *Acacia* forest, *Euphorbia* scrub, and habitat around water bodies (Fig 4). At the 3^rd^ order, within the 95% KDE, lions show preference for the grassland and mixed forest, while avoidance for the *Acacia* forest, *Euphorbia* scrub, scrubland, and habitats around water bodies (Fig 4).

**Fig 5:**
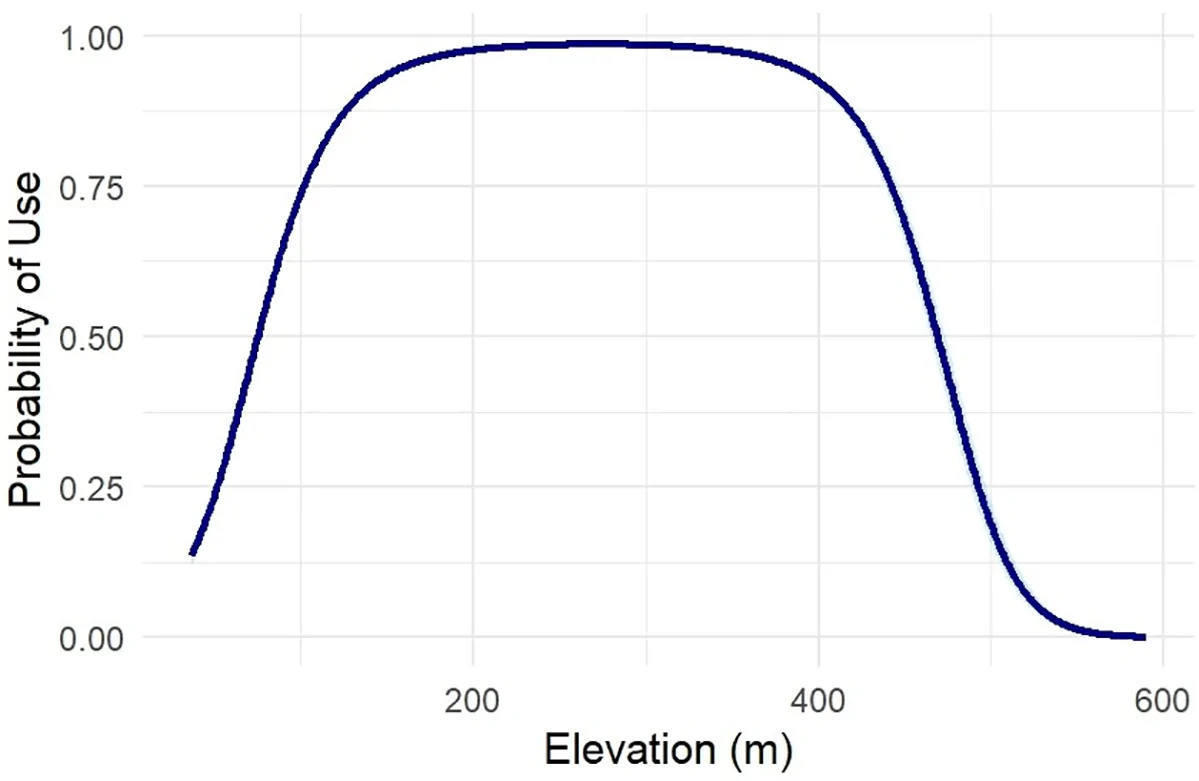
Use of elevation by lions in Barda Wildlife Sanctuary, Gujarat, India. The blue line in the graph indicates predicted use.

A quadratic logistic regression was employed to evaluate the effect of elevation on lion space use. Results revealed that both the linear and quadratic elevation terms were highly significant (p < 0.001), indicating a non-linear response to elevation. Lions showed a sharp increase in use of lower elevations, with probability of use rising rapidly up to mid-elevations (approximately 350–400 m; Fig 5; Fig S6 in S1 File). Beyond ~400 m, the probability of use no longer increased and instead reached a plateau, indicating a saturation effect where higher elevations do not provide additional ecological benefits for lions rather than an active preference for steeper or higher terrain. This pattern suggests that lions preferentially utilise mid-elevation zones, whereas higher elevations offer no additional benefit, likely due to variations in terrain, vegetation structure, or resource availability.

### Prey availability and diet pattern

Based on estimates from Ram et al. (2024) [15], prey availability in Barda is low and skewed towards the wild pig (*Sus scrofa*), which has the highest prey density (individuals/km^2^) at 10.77 ± 4.59, followed by nilgai (*Boselaphus tragocamelus*) (3.50 ± 0.51), Indian hare (*Lepus nigricollis*) (3.03 ± 0.73), and spotted deer (*Axis axis*) (0.62 ± 0.04). Diet pattern analysis revealed that lions consumed six prey species, of which three were wild—namely, wild pig, spotted deer, and nilgai—while the other three—buffalo, cow, and camel—were domestic prey species. Wild prey contributed (PO) approximately 72% to the diet, whereas domestic prey contributed 28% (Table 2). Among wild prey, wild pigs accounted for the largest share at 55%, while among domestic prey, cow accounted for the most at 17%. In terms of biomass consumption, wild pigs accounted for 47%, indicating a strong reliance of lions on wild pigs in Barda, with domestic cows contributing 20%, the next highest after wild pigs (Table 2).

**Table 2:** Diet pattern of the Asiatic lion in the Barda Wildlife Sanctuary.

Prey Species	Count of Occurrence	Percent Occurrence	Biomass Consumed	Percent Biomass Consumed
Wild Pig	36	55	115.56	0.47
Domestic Cow	11	17	48.07	0.20
Nilgai	8	12	34.96	0.14
Domestic Buffalo	6	9	26.22	0.11
Spotted Deer	4	5	14.68	0.06
Domestic Camel	1	2	4.42	0.02

### Prey augmentation

In Barda, two prey species, namely spotted deer and sambar, have been augmented through a captive breeding programme since 2007 and translocation from Gir Protected Areas since 2024. In the breeding programme, a total of 195 spotted deer were brought from two zoos, Sakkarbaug Zoological Park in Junagadh and Saiyajibaug Zoo in Vadodara, Gujarat, while 348 were born at the breeding centre in Barda between 2002 and 2023. A total of 326 spotted deer were released into Barda from 2007 to 2023, including 133 adult males, 187 adult females, and six fawns. For sambar, 26 individuals were brought from Sakkarbaug Zoological Park, Junagadh; Kamla Nehru Zoological Garden (Kankariya Zoo), Ahmedabad; and Indroda Nature Park Zoo (Gujarat Ecological Education and Research Foundation), Gandhinagar, between 2004 and 2014. A total of 38 sambars were released from the captive breeding centre between 2014 and 2023, comprising 17 adult males, 20 adult females and a fawn [20]. Additionally, to augment the wild population in Barda, the Gujarat Forest Department has initiated a project to translocate spotted deer and sambar from the Gir Protected Areas to Barda after due approval from the competent authority. As part of this project, 400 spotted deer and 2 sambars were captured using Boma techniques and released between June 2024 and January 2025.

## Discussion

Conservation of the naturally dispersed or augmented population of large carnivores from their source population requires effective conservation of the habitats present in the population buffers for their long-term survival [13]. Barda is one of the crucial population buffers for the lion dispersing from the south-western side of Gir and is therefore vital for their long-term conservation. The present study assesses key ecological and social aspects for conserving and managing lions in Barda.

### Space use and habitat utilisation

This study indicates that the average home range sizes of lions in Barda are comparatively smaller than those observed in multi-use landscapes but larger than estimates reported from the Gir Wildlife Sanctuary and National Park [38,39]. Variations in home range dimensions across different habitats are likely driven by ecological and anthropogenic factors, including prey abundance, habitat heterogeneity, and levels of human disturbance [40–42]. For a long time, Barda has experienced significant anthropogenic pressures, including grazing and the reliance of local communities on fuel wood, fodder, and grasses [20]. Elevated grazing intensity and resource extraction have potentially diminished prey availability, contributing to declines in prey populations and habitat degradation. This prey scarcity may have influenced lions to expand their home ranges compared to those in habitats like Gir, where higher prey densities facilitate smaller territorial extents; however, as prey availability was not directly measured in the present study, this interpretation should be considered as a plausible explanation supported by existing literature rather than a directly tested relationship. In addition, the high elevation and rugged, hilly terrain of the landscape likely constrain movement efficiency and resource accessibility, requiring lions to range over larger areas to meet their energetic needs. This topographic complexity may therefore contribute to larger home ranges compared to Gir, where relatively larger flatter terrain facilitates more efficient movement and allows lions to fulfill their requirements within a relatively smaller area.

Additionally, our analysis of female lion home ranges revealed variability, with individual AF_3 exhibiting the largest home ranges at both 95% and 50% utilisation distributions, while SAF_2 showed the smallest. Previous research suggests that lactating females tend to have reduced home ranges due to energetic and protective demands associated with cub rearing [43–45]. During the study period, SAF_2 was observed with dependent cubs, which likely limited its movement and resulted in smaller home ranges than those of other individuals. Conversely, the male lion SAM exhibited a comparatively smaller home range, in contrast to earlier findings that sub-adult males tend to have larger spatial extents due to dispersal behaviour and territory establishment [39,46–48]. SAM was the only male lion in the study area; therefore, the absence of competing males may reduce dispersal pressure typically observed in sub-adult males, resulting in more restricted space use and smaller home ranges.

This study demonstrates that lions exhibit greater overlap in their home ranges (95% KDE) compared to their core areas (50% KDE). This pattern is consistent with previous findings [30,49,50] where large carnivores tend to show greater overlap in their broader home ranges than in their core areas. Core areas are areas of exclusive use for vital activities such as resting, cub rearing, prey sourcing, and kill sites, which account for the reduced overlap observed in these areas [1,51].

The proximity values recorded among radio-collared lions in Barda demonstrate varying degrees of spatial association, with some individuals showing strong spatial overlap while others exhibit low proximity. While these patterns provide useful insights into spatial associations among individuals, they should be interpreted cautiously, as proximity-based metrics alone do not fully capture social structure without supporting behavioural or long-term data. These findings align with previous studies indicating that spatial proximity among lions is influenced by factors such as kinship, reproductive status, resource availability, and territorial behaviour [52,53]. The notably low proximity indices observed between certain females, such as AF_1 with AF_3 and AF_2, suggest a tendency toward exclusive space use. Earlier research [52,54,55] on both Asiatic and African lions has documented behaviours within prides where dominant females maintain distinct ranges to reduce intra-group competition. Such low spatial interactions indicate a strategic adaptation aimed at minimising resource overlap, thereby enhancing individual fitness and promoting pride stability in competitive environments.

In contrast, the proximity indices revealed that the strongest associations were between specific female dyads, such as AF_2 and SAF_1, as well as AF_3 and SAF_1, possibly indicating cooperative behaviours related to maternal support or protection. Previous research on African lions has shown that related females tend to form strong spatial associations to assist with cub rearing and cooperative hunting [45,56]. In Barda, where prey density is lower than in Gir PAs, this association may also function as a strategy to enhance hunting efficiency. Additionally, moderate proximity indices observed between sub-adult male and adult females could suggest a transitional phase, wherein younger male establish spatial associations prior to dispersal. Funston et al. (2003) [57] documented that sub-adult males in African lions form temporary coalitions before either joining new prides or becoming nomadic. The low associations between SAF_2 and SAM align with prior findings that sub-adults exhibit partial social bonds as they navigate towards independence [45]. This weak association might also result from SAF_2’s protective behaviour, as this individual was caring for dependent cubs. The minimal association between AF_1 and SAF_2 indicates a tendency toward spatial segregation. Consistent with earlier studies on both Asiatic and African lions, younger females either integrate into existing prides or establish new groups depending on habitat suitability and pride structure [52, 58].

Large carnivore habitat use is influenced by key drivers, including prey availability, vegetation structure, intra- and interspecific competition, and human disturbance [59–61]. Jacobs’ index revealed a scale-dependent habitat preference in lions within Barda Wildlife Sanctuary, distinguishing between 2^nd^-order (home range selection) and 3^rd^-order (within-home range selection) habitat use. At the home range level, the 95% KDE lion showed a preference only for mixed forest. In Barda, mixed forests occur at moderate to high elevations and experience lower levels of human disturbance than flatter habitats, such as *Acacia* forests. The higher tree diversity in mixed forests likely supports greater prey abundance and offers shaded, secure resting sites during the hottest parts of the day. These factors collectively contribute to a preference for mixed forests when establishing home ranges, underscoring their significance as favourable habitats for lions within the sanctuary.

In contrast, the habitats avoided by lions—*Acacia* forest and *Euphorbia* scrub—are located in flatter regions, particularly along the northwestern and northeastern edges of the Barda, where human activity is more intensive. Within core areas, specifically the 50% KDE and 95% KDE home ranges (3^rd^-order), lions preferentially used grasslands and mixed forests. Although grasslands are limited to small patches at medium to high elevations, field observations indicate that lions frequently rest in these areas because of their relatively flat terrain, which provides comfortable resting sites with good visibility. These open, elevated locations enable lions to rest while maintaining visual surveillance of their surroundings, thereby reducing disturbance and increasing safety for both adults and cubs.

Further behavioural strategies for thermoregulation in lions may also account for their preference for open habitats, such as grasslands, especially at higher elevations, where temperatures tend to be cooler. Lions frequently rest in open or semi-open areas with stronger airflow, which facilitates cooling and helps mitigate heat stress during warmer periods [62]. Open habitats like elevated grasslands provide better natural ventilation than dense forests, where stagnant air and higher humidity can impede heat dissipation. Collectively, these mechanisms indicate that selecting grassland patches—characterized by wind exposure, enhanced airflow, and lower humidity—constitutes a significant behavioural thermoregulatory strategy, enabling lions to avoid heat stress while resting or surveying their core areas.

The binary quadratic regression analysis indicated that lion use increases at lower elevations, peaks at mid-elevations, and declines beyond approximately 400 meters. This pattern reflects a combination of ecological and behavioural mechanisms influencing elevation use in Barda. Mid-elevation zones in Barda are characterised by mixed forests and grassland patches that provide favourable resting sites, prey availability, and reduced human disturbance compared to the more accessible lowlands. Similar elevation-related behaviour has been reported in Asiatic lions of the Girnar Wildlife Sanctuary, where lions frequently occupy low- to moderate-elevation areas [63]. In the Greater Mapungubwe Transfrontier Conservation Area in Africa, elevation was identified as a key predictor of lion space use, partly because protected habitats are concentrated within specific altitude bands. In Barda, mid-elevation areas include rocky outcrops and uneven terrain that may offer elevated vantage points for lions, comparable to observations from the Serengeti plains where such features enhance prey detection [64,65]. In Barda, mid-elevation zones offer thermal benefits such as cooler temperatures, increased airflow, and lower humidity, as well as structural complexity for concealment and reduced exposure to human activity. The decline in use beyond 400 meters is likely attributable to the higher energy expenditure required for traversing this elevation, which probably outweighs the benefits compared to elevations between 200 and 400 meters.

### Prey availability and diet pattern

Prey availability in Barda is currently limited and skewed towards wild pigs, which dominate the diet of lions in the area. This supports our hypothesis that lions depend on the numerically abundant prey. Previous studies in protected areas in India, such as Gir, indicate reduced reliance on wild pigs due to the presence of alternative prey, including spotted deer, sambar, and nilgai [66,67]. When prey availability shifts, large carnivores tend to switch from their preferred prey to more abundant species [68,69]. Hayward and Kerley (2005) [70] found that lions typically prefer prey weighing between 200 and 250 kg, as their social hunting strategy allows them to handle larger prey more effectively than solitary predators. The high contribution of wild pigs to the lion diet, despite these wild pigs falling outside the preferred prey weight range, may be due to the comparatively low availability of large prey such as sambar and nilgai. Notably, nilgai were the second-most-consumed prey species in Barda, suggesting that lions can prey on large animals even when they are scarce. However, the observed prominence of wild pigs may also be attributed to seasonal variation in prey availability and detectability, as well as the short duration of the present study. Therefore, long-term monitoring encompassing multiple seasons is necessary to assess prey preferences accurately and to distinguish seasonal effects from true dietary specialization.

In Barda, domestic cattle, primarily cows and buffaloes, constitute the predominant prey species for lions. Consistent with previous findings, lions tend to prefer large-bodied prey, and domestic cattle, due to their substantial size, offer a significant biomass reward [70]. Furthermore, the lack of anti-predator strategies among domestic cattle increases their susceptibility to predation [67]. The historical absence of lions in Barda may have led to reduced vigilance in domestic cattle compared with populations in areas such as Gir, where lions have been present for longer. Consequently, the combination of large body size and reduced anti-predator responses, resulting from the long-term absence of lions, positions cattle as a key prey resource for lions in this area.

Prey augmentation is a vital strategy for conserving large carnivores [71,72], as prey abundance is a key determinant of their density, social structure, and livestock depredation patterns [73]. As previously noted, Barda has a lower prey base than other protected areas, such as Gir, with wild pig being the predominant species [15]. An increase in lion density in Barda, given limited wild prey availability, could lead to greater reliance on domestic livestock for sustenance. To mitigate conflicts, particularly livestock depredation, a prey augmentation program was initiated, introducing spotted deer and sambar—primary prey species for lions—to restore a functional predator-prey relationship and reduce dependence on domestic cattle. Moreover, wild herbivores, particularly large ungulates (>15 kg), play a vital role in terrestrial ecosystems [74]. They play essential ecological roles in maintaining ecosystem structure and function through nutrient cycling, soil structure, and succession [75,76]. Therefore, the translocation of herbivores in Barda will benefit both ecosystem restoration and carnivore conservation. However, the success of wild prey augmentation is highly contingent on habitat quality, carrying capacity, and interspecific competition among the reintroduced prey species [73,77]. The long-term viability of reintroduced populations of spotted deer and sambar depends on forage availability and quality, habitat conditions, and livestock populations.

The natural recolonisation of lions in Barda has initiated a series of ecological benefits, highlighting the role of charismatic species in conservation efforts. This phenomenon has prompted the forest department to enhance protection and conservation measures in the sanctuary. Notably, most of the vacant frontline uniformed staff posts have been filled on a priority basis, and eight full-time lion trackers have been commissioned to monitor lion movements and health daily, facilitating rapid response and reducing human-wildlife conflict.

Significant advancements in management infrastructure have been implemented, including enhancements to the wireless communication network to facilitate real-time coordination among frontline staff, the establishment of new forest check-posts and barriers to increase surveillance, and the initiation of regular training programs to build the capacity of field personnel for better lion conservation in Barda. Habitat improvement strategies, such as waterhole management and the removal of invasive species, have contributed to increased prey availability and overall habitat quality. Moreover, the introduction of more efficient, streamlined compensation mechanisms has ensured prompt claim resolution, thereby fostering greater trust and cooperation with local communities.

Third, ecotourism was started in Barda in 2024, involving local communities as nature guides and safari vehicle owners and operators. This initiative has introduced alternative economic opportunities and promoted positive attitudes towards lion conservation. Additionally, residents are engaging in habitat management activities, including the removal of invasive species, the restoration of degraded areas, and the making of firebreaks (15 meters on both sides of the forest mud tracks) to mitigate wildfire risk within the sanctuary. Local residents are also employed at check posts, watch towers, forest setups, in protection works, and other similar works and facilities. These initiatives enhance collaborative stewardship between management authorities and local communities.

These advancements collectively illustrate an enhanced commitment across social, ecological, and institutional domains to the long-term conservation of lions within Barda and to augmenting the sanctuary’s ecological resilience.

In a nutshell, this study provides valuable insights into the ecological and social dynamics of Asiatic lions in Barda, which were reestablished after about 144 years, aiding the development of better conservation and management strategies. Results show that lion home ranges in Barda are larger than those in Gir but smaller than those in multi-use landscapes, likely due to reduced prey availability, anthropogenic pressures, and terrain characteristics. Lions exhibited significant overlap in their home ranges and core areas, indicating territorial behaviour. Habitat selection varied by scale, with lions preferring mixed forests at the home range level and grasslands and mixed forests within the core and home ranges. Diet pattern analysis revealed that wild pig is currently the primary prey, with limited consumption of spotted deer, sambar, and nilgai, highlighting the need for continued efforts to augment the wild prey base in Barda.

### Implications for conservation and management

At the landscape management level, lions currently exhibit limited overlap in 95% home ranges and minimal overlap in core areas, likely due to their low population in Barda. However, future population increases may potentially heighten intraspecific competition. The lion population in Barda has grown to 17 individuals, which may intensify competition for space. As the population continues to expand, dispersal into surrounding habitats is likely, based on observed patterns in other lion populations, highlighting the need for a landscape-level conservation and management strategy. Identifying potential habitats in nearby areas and corridors, and improving habitat connectivity, will support natural dispersal and accommodate the growing and dispersing populations. Additionally, monitoring lion movements beyond Barda is crucial for reducing human-lion conflicts in the multi-use landscape.

Regarding habitat selection, lions in Barda show specific preferences across multiple spatial scales. At the second-order level, they strongly prefer mixed forests. At the third-order level, within core areas, lions prefer grasslands and mixed forests. These findings underscore the importance of woodland and open habitats in Barda and the need to prevent their degradation. Additionally, open areas at mid- to high elevations (200–400 meters) are important components of lion habitat use and warrant targeted conservation efforts.

Regarding prey augmentation, spotted deer and sambar populations are ongoing and monitored accordingly. However, ongoing augmentation and long-term monitoring are vital for assessing survival, reproduction, and ecological impacts. The continued survival of these prey populations in the natural environment is critical for ecosystem restoration, increasing the wild prey base for lions, and reducing livestock depredation and thereby conflicts. Future studies on ecosystem functioning, restoration work, diet patterns, habitat changes, and the effectiveness of management and protection measures will provide valuable insights into the conservation and management of Barda Wildlife Sanctuary.

## Supporting information

S1 File Photographic plates depicting different habitat types in Barda Wildlife Sanctuary and their utilization by lions. The photographs were captured by the first author (Mohan Ram) for the paper titled “Beyond Gir: Barda Wildlife Sanctuary as a potential stronghold for Asiatic lions” and are licensed under the Creative Commons Attribution License (CCAL) CC BY 4.0.
(PDF)
