# Supplementary material for: Beyond Gir: Barda wildlife sanctuary as a potential stronghold for Asiatic lions
Source: PLoS One. 2026 Sep 9;21(9):e0354096. doi: 10.1371/journal.pone.0354096 (PMC13557388; doi:10.1371/journal.pone.0354096)
Supplement: Supplementary file 1 [file pone.0354096.s001.pdf]

Photographic plates depicting different habitat types in Barda Wildlife Sanctuary and their utilization by lions.

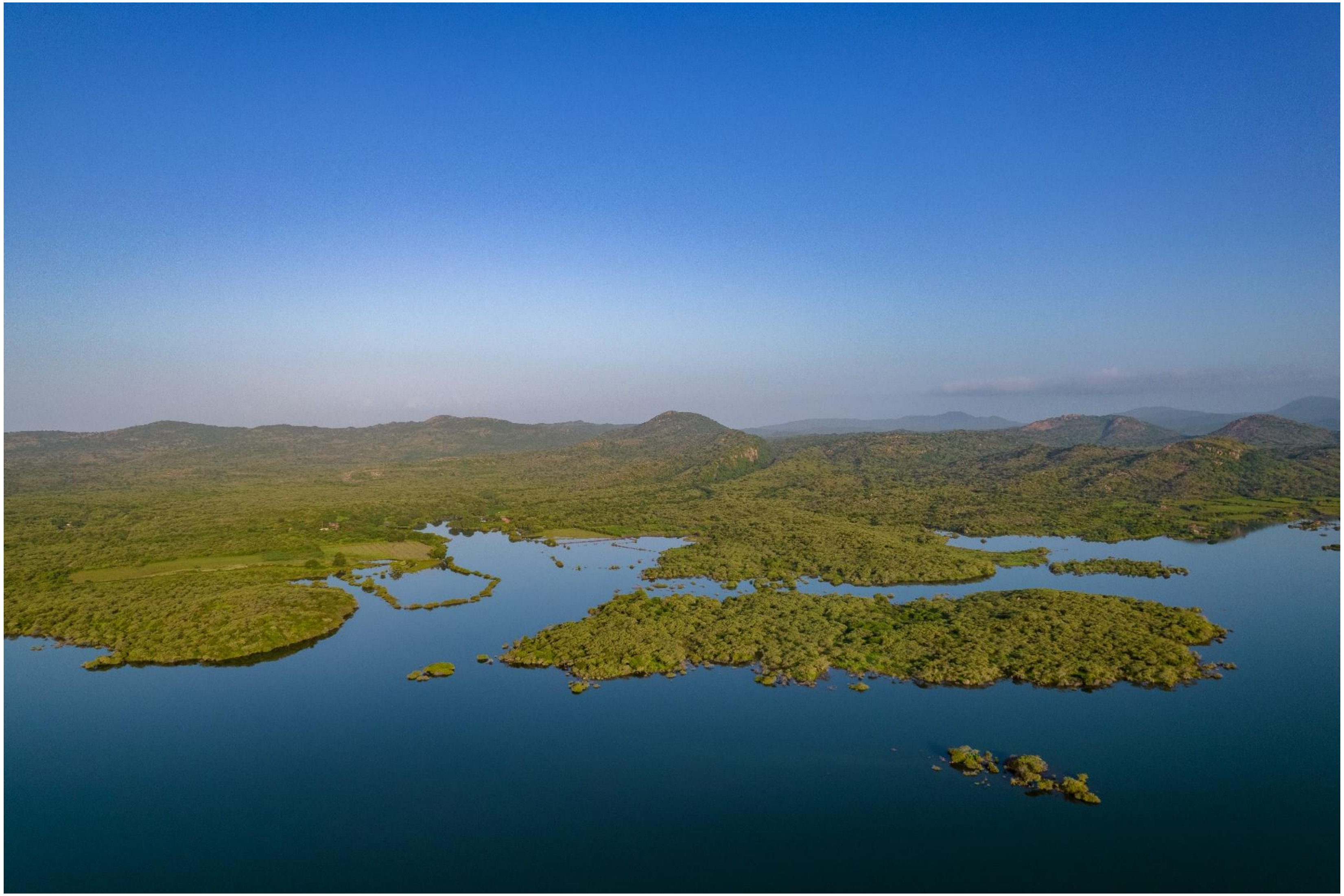

Fig. S1: Aerial view of Barda Wildlife Sanctuary (Barda WLS) representing landscape features and habitat types including large water bodies and undulating and hilly terrains.

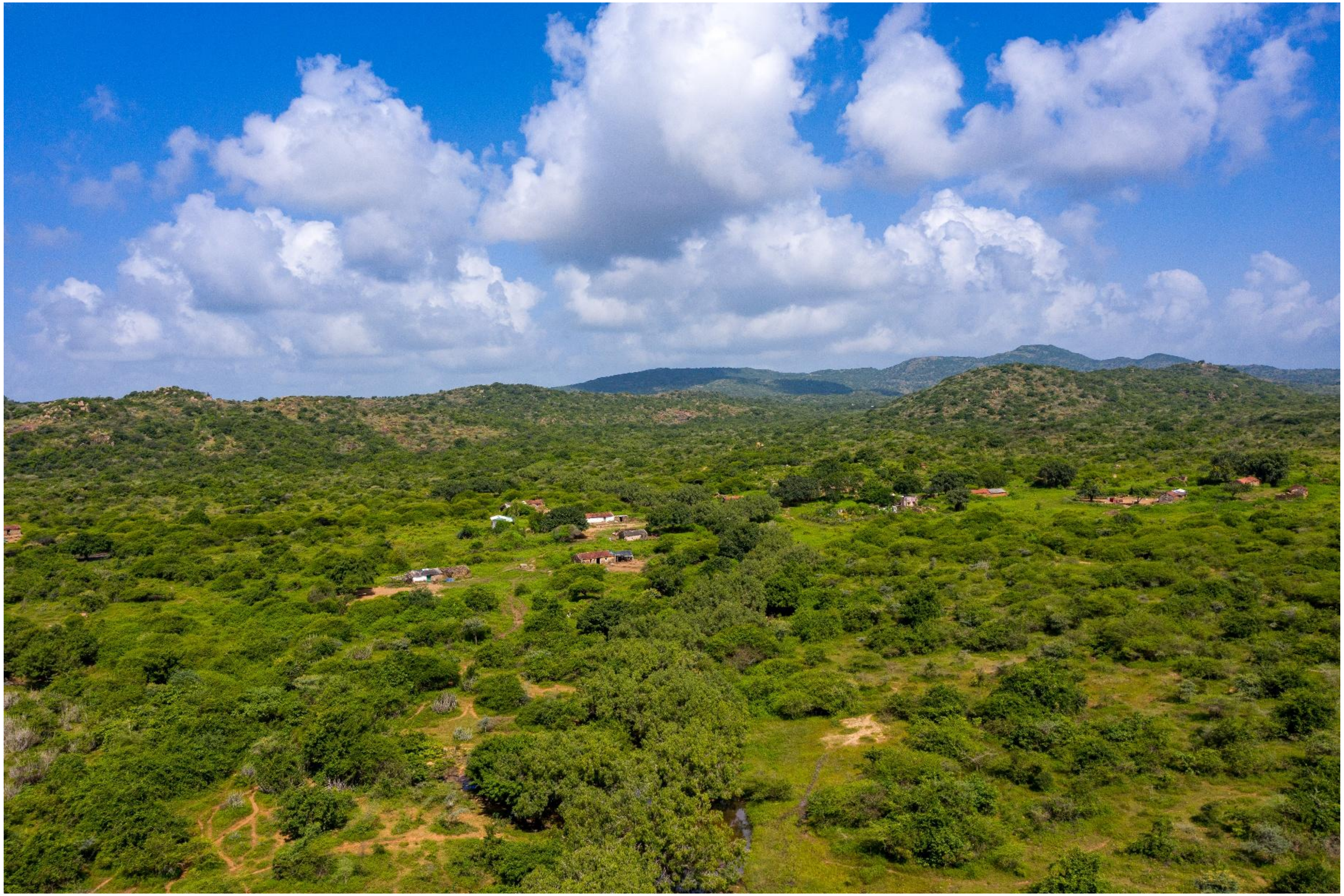

Fig. S2: A landscape view of Barda WLS showing the undulating terrain, mixed forest patches and open habitat with human habitation in flat terrain.

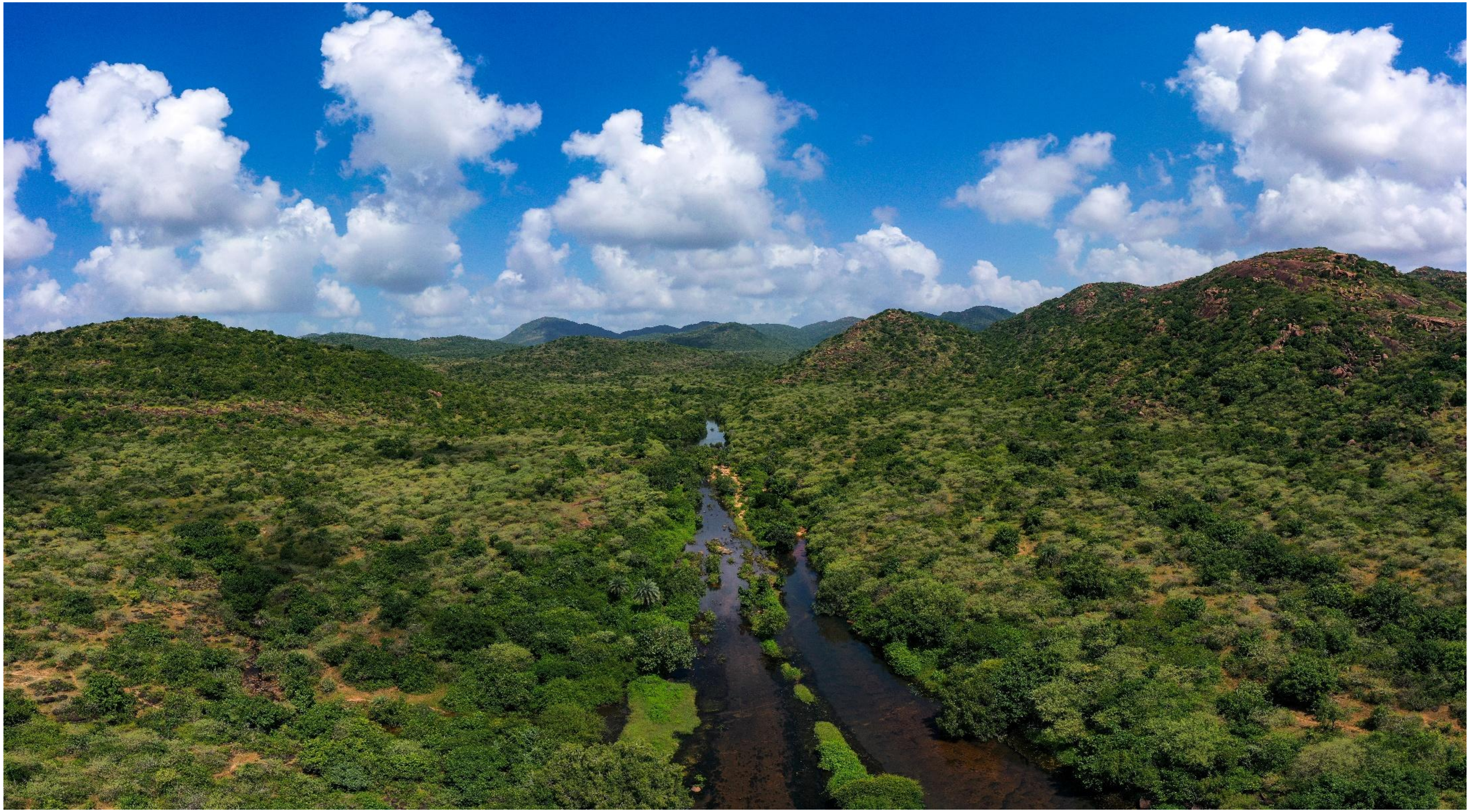

Fig. S3: Riverine landscape with water and surrounding vegetation in Barda Wildlife Sanctuary, representing available habitat and crucial resources that provide cooler conditions and summer shelter for lions.

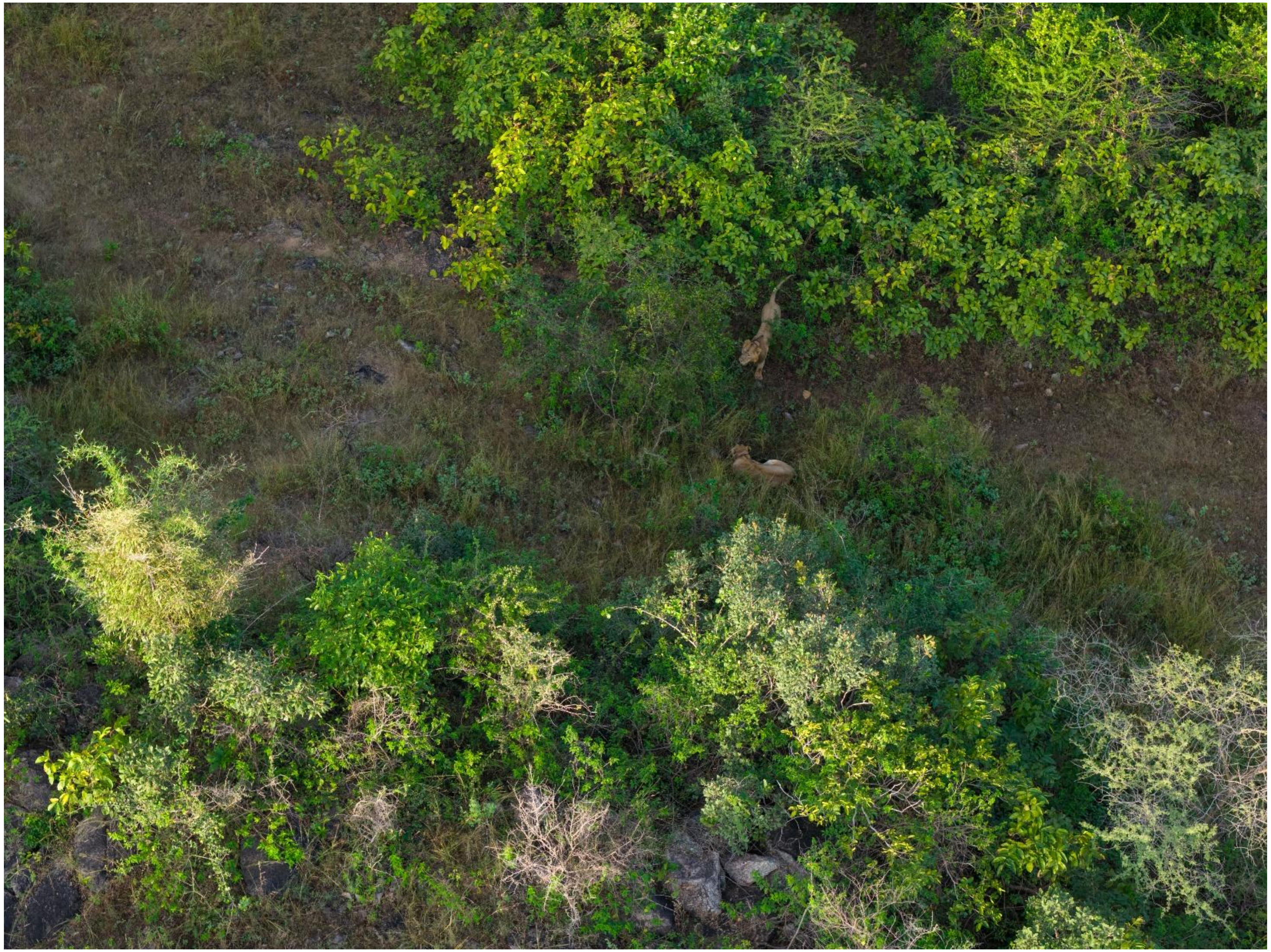

Fig. S4: A male and female lion interacting within the mixed forest habitat of Barda WLS, demonstrating habitat use by the species.

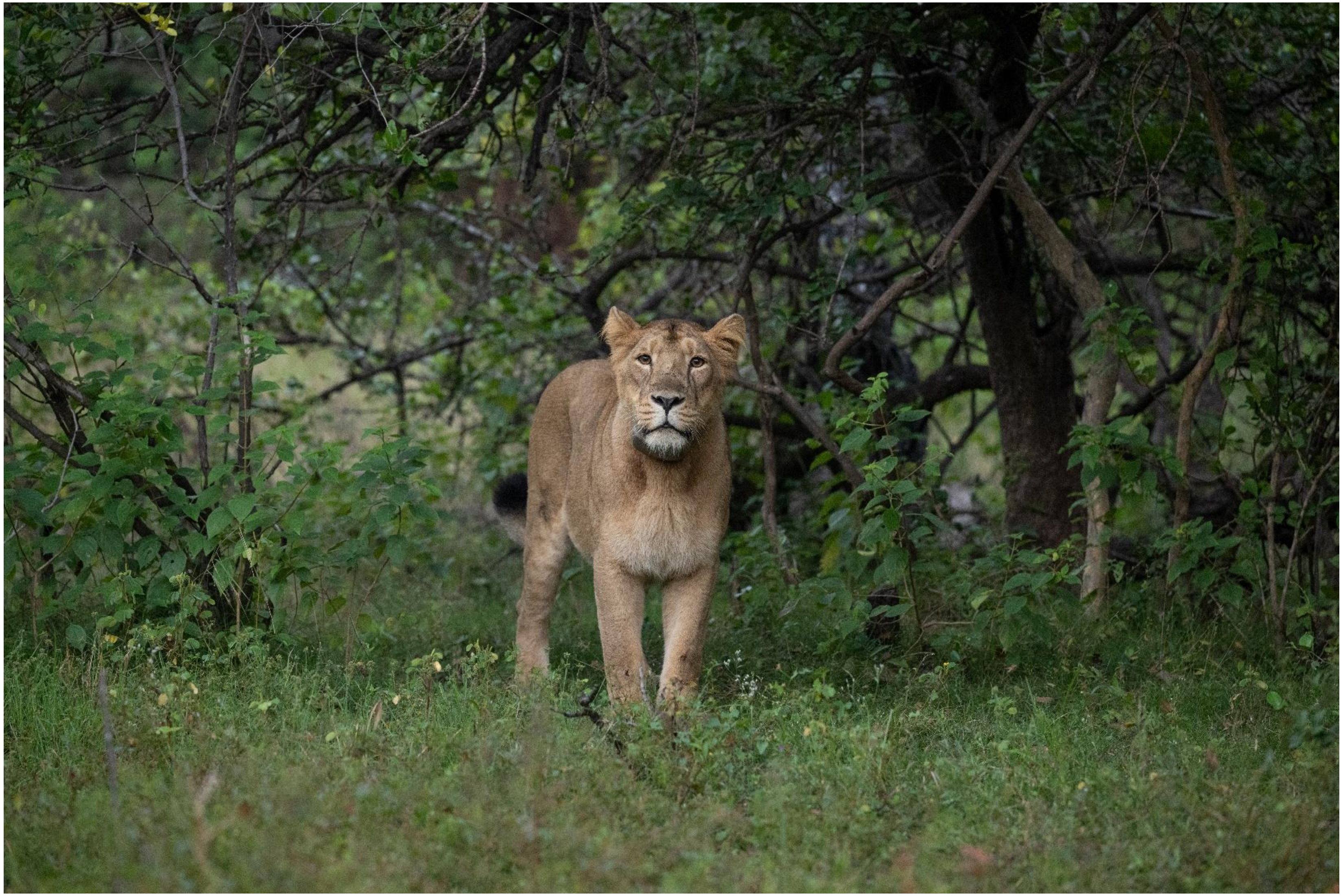

Fig. S5: Radio-collared female using mixed forest habitat in Barda WLS.

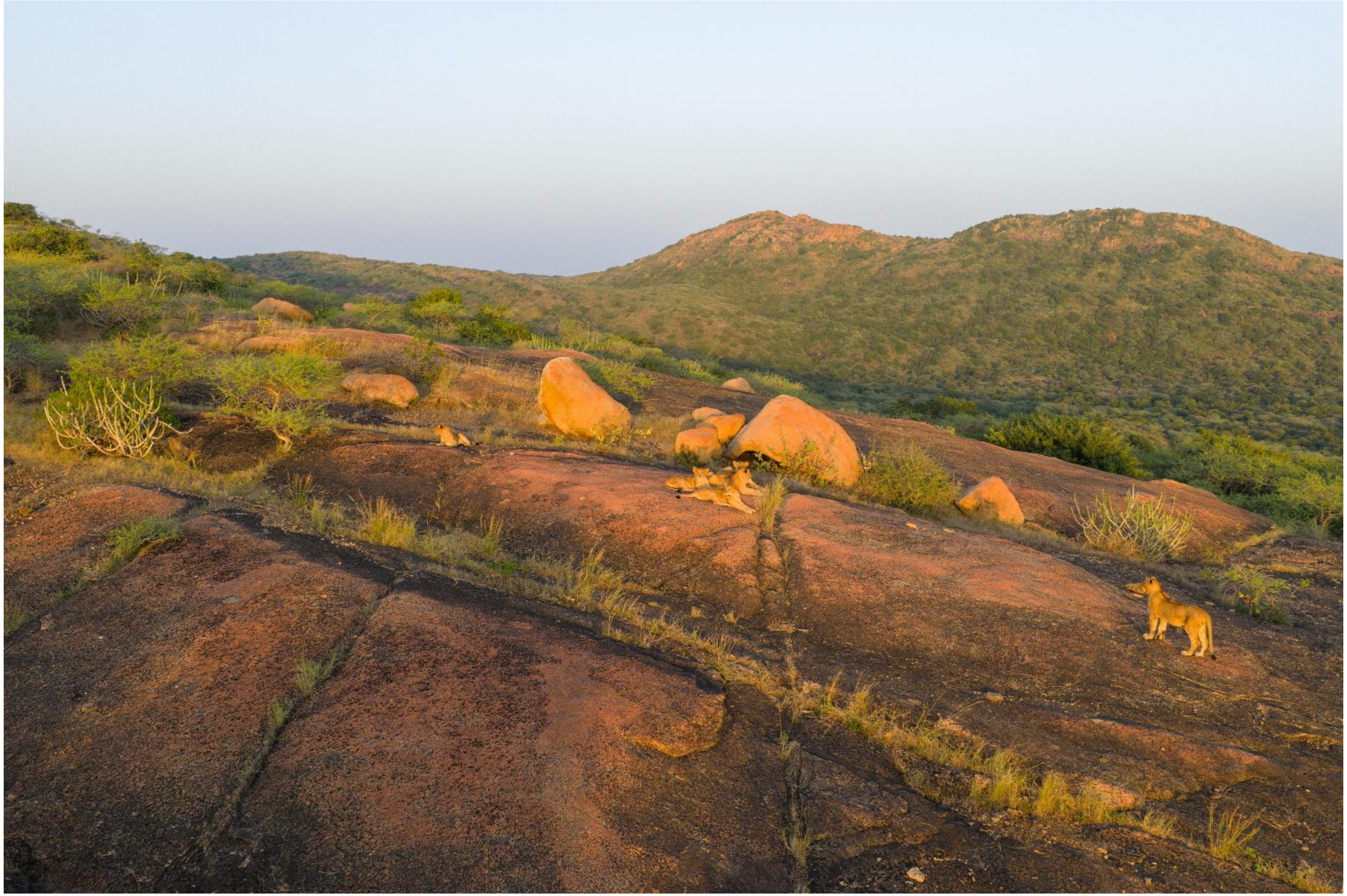

Fig. S6: A lion pride using the rugged, hilly open habitat of Barda WLS, highlighting successful recolonization and the species' effective use of hilly slopes.

*****
